# Evaluating the Application of Portable Handheld X-ray Fluorescence (XRF) Scanner for Determining Seafood Provenance: A Case Study on *Penaeus monodon*

**DOI:** 10.3390/foods12152874

**Published:** 2023-07-28

**Authors:** Nondita Malo, Debashish Mazumder, Jagoda Crawford, Patricia Gadd, Karthik Gopi, Neil Saintilan, Jesmond Sammut

**Affiliations:** 1 Centre for Ecosystem Science, School of Biological, Earth and Environmental Sciences, The University of New South Wales (UNSW), Sydney, NSW 2052, Australia; 2 Australian Nuclear Science and Technology Organisation (ANSTO), Locked Bag 2001, Sydney, NSW 2232, Australia; 3 Sydney School of Public Health, The University of Sydney, University Centre for Rural Health, Sydney, NSW 2052, Australia; 4 School of Natural Sciences, Macquarie University, Sydney, NSW 2109, Australia

**Keywords:** portable X-ray fluorescence (pXRF), seafood, provenance, authentication, traceability

## Abstract

Seafood elemental profiling (SEP) is the quantification of a range of elements in seafood products and may serve in addressing questions of seafood provenance and quality. Traditional methods for analyzing soft tissue present several limitations for the industry-level use of SEP. Portable handheld X-ray fluorescence (pXRF) analysis is a promising alternative to conventional methods; however, its application for biological analysis has not been fully established. Intact giant tiger prawn (*Penaeus monodon*) abdomens were analyzed with a Vanta M series XRF portable analyzer following a novel soft tissue protocol. Exploratory statistics (principal component analysis, nonmetric multidimensional scaling, and canonical discriminant analysis), as well as random forest models, have been implemented with pXRF profiles, yielding 81% accuracy when assigning the geographical origin of *P. monodon*. The results of this study highlight that SEP via pXRF is a viable industry-level analysis, and its application will depend on improved instrument calibration to account for fluctuating wetness factors that are influenced by cooking, storage, and other pre- and post-harvest treatments.

## 1. Introduction

Seafood elemental profiling (SEP) is the quantification of a range of elements in a seafood product or sample, representing a tool that has gained increasing interest from seafood industry stakeholders [1]. Profiling assists in food quality control measures and ensures consumer safety by focusing on the heavy metal readings of the profile [2] (A profile is unique and may act as a product signature or fingerprint, which can then serve in authenticating products and detecting food fraud along the supply chain. [3]). The conventional methods for SEP are numerous and typically use hyphenated inductively coupled plasma mass spectrometry (ICP-MS) techniques [4,5,6], stable isotope analysis (SIA), and fatty acid profiling [7,8,9,10,11]. These methods have been the main approaches for answering seafood regulatory questions about the presence of toxins and the aspects of authenticity like provenance (geographic origin and production method of the seafood product [12]). The reliability of these methods comes with several disadvantages from an industry standpoint, including lengthy sample preparation, digestion, and processing time. 

Regulation and decision-making require quick access to an array of high-quality data in order to address food fraud and ensure seafood safety [13]. In essence, for seafood industry stakeholders to capitalize on SEP’s full utility, the analytical process must be expedited with simple and in situ modes of data capture to quickly and reliably analyze numerous samples. Procedures for the conventional ICP-MS techniques offer minimal room for adaptation. The potential of other instruments to hasten the analysis for SEP were investigated by Gadd et al. [14] and a soft tissue elemental profiling protocol using X-ray fluorescence (XRF) analysis via an Itrax μXRF core scanner was established. Though this instrument is typically used on media with denser matrices like rock and soil core samples [14], the XRF analysis reduces the time needed to perform SEP from hours to minutes per sample. While the analysis by Gadd et al. [14] involved sample drying and powdering, here, we investigate the potential of analyzing fresh samples. Unlike ICP-MS, using XRF on fresh samples has the added benefit of being a non-destructive analysis requiring no sample digestion [15], which is ideal in practice, as samples are persevered and can be returned to the supply chain or used for additional analyses.

Provenance is an intricate issue that can be tackled using SEP, because the multiple elemental measurements together encapsulate more information about the organism’s unique geographic settings, ambient water qualities, and alimentation [16,17,18]. Studies have examined the use of various elements, such as Li, Al, V, Fe, As, Rb, Sr, Cd, Pb, Ce, Nd, Pr, Sm, Eu, Gd, Tb, Dy, Ho, Er, Tm, Yb, Lu, Th, and Y [19,20,21,22]. Gopi et al. [20,23] trialed the protocol of Gadd et al. [14] by profiling 31 elements in Barramundi (*Lates calcarifer*) and giant tiger prawns (*Penaeus monodon*). The SEP was then implemented in a linear discriminant analysis (LDA [24]) and random forest (RF [25]) machine learning models to determine the provenance of the two species and reported high degrees of accuracy. The results of these studies emphasize the effectiveness of SEP via XRF and its potential utility in assessments that are integral to the industry’s fight against provenance-related food fraud. Despite its time efficiency and reliability, Itrax is lab-based, with limited accessibility, and requires instrument scientists to process samples; this presents issues similar to those of conventional SEP methods. In 2022, the instrument was valued at over USD 700,000, and analyses cost USD 500 per meter of scanned material. Hence, the issues with accessibility, expenses, time, and labor associated with this instrument make SEP with Itrax unfeasible when dealing with the high volumes of samples used in seafood regulation.

A portable X-ray fluorescence (pXRF) analysis offers a solution to the aforementioned limitations of Itrax, because pXRF utilizes a more affordable, mobile, and user-friendly analytical instrument [26]. In common with lab-based XRF, portable XRF is rapid and hitherto been mainly used for geological and pedological analyses [15]. Soft tissue analysis via pXRF has recently gained the attention of those looking to improve in-field soft tissue assessments in edible plants, but most of these exploratory studies relied on powdered samples or the removal of moisture to some degree [25,26,27,28,29,30,31], as a high moisture content and lack of density impedes fluorescence detection [32]. This body of research shows that pXRF has great potential as a standard, in situ, biological analysis; however, the existing protocols lack an emphasis on the speediness necessary for industry use.

The main objectives of the present study were to establish a soft tissue analysis method for a pXRF instrument and to determine if a pXRF instrument can be used on the market floor or at any point in the seafood supply chain. Giant tiger prawns (*Penaeus monodon*) were used to establish the analytical method due to their popularity and economic value in the seafood industry [33]. The intention of the method is to allow seafood industries and regulatory bodies to meet the traceability challenges with a high-impact tool for maintaining integrity in the supply chain. 

## 2. Experimental Methods

### 2.1. Materials

#### 2.1.1. Analytical Instruments

An Olympus Vanta M series XRF portable analyzer with a 4W rhodium anode X-ray source and large area silicon drift detector was used to develop the pXRF soft tissue protocol. Vanta is designed to be handheld; however, the analyses for this study were conducted with an accessory benchtop workstation to stabilize the instrument and limit the operator’s exposure to radiation. Ion beam analysis (IBA) was used to calibrate Vanta for the scanning of powdered samples, as both instruments are measured in parts per million (ppm), making for a more direct comparison. The IBA used for Vanta’s calibration was conducted using the Lucas Heights, Australian Nuclear Science and Technology Organization’s 2MV STAR Tandetron Accelerator. 

#### 2.1.2. Sample Collection and Preparation

Giant tiger prawns (*Penaeus monodon*) were used in this study not only due to their economic value in the seafood industry [33] but also to build on the earlier provenance research using carbon- and nitrogen-stable isotopes and elemental profiling through Itrax *P. monodon* research [12,14,21]. Frozen giant tiger prawn specimens of known origin were provided by Sydney Fish Market Pty Ltd. (Pyrmont, NSW, Australia). Prawns were sourced from 6 known sites across the Australian east coast, with representation from both farms and wild fisheries. One site had samples taken during both January 2021 and February 2021, and another site had samples that were cooked and uncooked, making 8 groups total, with 5 prawns in each group (*n* = 40) (Table 1).

For calibration with the IBA and repeatability analysis, powdered samples were made by isolating a 5 cm^3^ section of abdominal tissue from the ventral side of the prawns, drying the sections in an oven at 60 °C for 48 h, and then grinding them until homogenous. XRF elemental analyses were conducted using thawed, minimally processed, and intact *P. monodon* abdomen: the shell and head were removed, while the hindgut and gonads were left in place. Prawns were pre-cooked in a blanch unless stated otherwise. The use of powdered samples for the repeatability and calibration analyses was deemed appropriate, as the results were purely used to assess the instrument and not part of the in situ pXRF protocol (analysis method) this study aimed to produce. During the analyses of the intact prawns, measurements were taken on the lateral right side of the abdomen in the middle of the first segmenting line—the thickest point on the prawn (2–3 cm). Using the largest cross-section of a biological specimen is always preferable, as a lack of density makes the soft tissue matrix a less than infinite thickness, in turn causing X-rays to scatter too far for the sample [34,35].

### 2.2. Protocol Methodology

The ideal exposure time to accurately quantify the elements while conserving time was determined with a comparison of a series of timed analyses. To create a protocol that minimizes sample preparation and, again, to save seafood industry stakeholders’ time, we used the minimally processed prawns to test different drying techniques; we hypothesized that varying degrees of wetness would affect detection, and a pre-analysis drying step might be required during sample preparation [32]. The repeatability of the analytical protocol was assessed to determine if Vanta pXRF produces consistent and reliable SEP.

#### 2.2.1. Validation of Methodology

##### Calibration and Arrangement of the Prawn Elemental Profile

Vanta calibration relied on a comparison of the elemental abundances detected following an established IBA protocol and those detected by Vanta pXRF for the same sample. Six powdered *P. monodon* samples were subjected to IBA following Grave et al.’s [35] proton-induced X-ray and γ-ray emission spectrometry (PIXE-PIGME) protocols. The same samples were then analyzed with Vanta under the factory default calibration settings. The corresponding elemental abundances (ppm) were plotted against each other in linear regressions, revealing Vanta’s over- or underestimations compared to the more precise results of IBA. The slope of the line of the best fit served as the element’s correction factor. Vanta was deemed calibrated for the giant tiger prawn analysis once the correction factors were established for all detectable elements in the powdered prawn samples. 

With Vanta calibrated to analyze *P. monodon*, preliminary analyses determined which elements the instrument successfully detected and quantified in the medium. An element was considered confidently detected and quantified and, thus, part of the prawn elemental profile if the error associated with the reading was less than one-third of the concentration. This was determined to be a reasonable cut-off by examining the results for the subsets of the data.

##### Repeatability

The findings of the calibration and exposure time assessments were implemented to then evaluate Vanta’s ability to generate consistent readings. Six powdered prawn samples were subjected to five pXRF exposures. The exposures were consecutive to avoid moving the samples and changing the point of analysis. Unprocessed prawn samples were not used for this assessment, as it was noticed that they retained heat during successive exposures, resulting in variable elemental detection. 

Vanta produced an error reading for each concentration, which provided some indication of repeatability; should the same analysis be run again, the concentration would deviate by a value equal to or less than this error. If Vanta is capable of producing replicate profiles, then an average of the 5 exposures’ instrument error readings (average_instrument_error) should account for the standard error observed between the 5 exposures’ concentration readings (i.e., if 𝜎 is the standard deviation of the *n* = 5 readings, $\mathrm{se}=\sigma /\sqrt{n}$). The delta value (Δ = average_instrument_error − se) compares these two errors and was calculated for each element of the SEP to determine the instrument’s ability to produce consistent readings. A positive delta value indicates that the standard error is comprised within the instrument error, and a negative value indicates that the standard error surpasses the instrument error, the latter scenario suggesting that the quantification and profile are less reliable. 

#### 2.2.2. Factors That Influence Measurements

##### Exposure Time

Vanta has three beams of different voltages (10, 40, and 50 kV) that enable the instrument to detect a large range of elements, as voltage is more or less needed to cause fluorescence, depending on the atomic mass of the element [16]. Users have the option of setting the exposure time for each beam, and to meet the objective of creating a time-efficient protocol, the exposure time should be set to the duration that reflects the minimum time required for optimal detection. Concurrently, the exposure time should also be long enough to minimize the error.

Whole giant tiger prawn samples (*n =* 5) were exposed to the three beams at the intervals of 15, 30, 45, 60, 75, and 90 s. The percent errors (100*error/concentration) for the element readings were calculated using Vanta’s concentration and error readings, and they were then plotted at their respective intervals to demonstrate the trend in errors over time. 

The slope improvement rate between two measurements (A and B) was used to infer the time when the percent error no longer improved and thus the optimal exposure time was achieved. The slope improvement rate was calculated by first taking the absolute difference between two consecutive slopes. This value was then divided by the absolute of the leading slope to determine the rate of improvement and multiplied by 100 to make a percent (i.e., 100*|Slope_at_A − Slope_at_B|/|Slope_at_A|). An acceptable time–error ratio was achieved when the slope no longer improved by at least 33%.

##### Sample Moisture

The effects of moisture are inevitable when analyzing an inherently wet medium; therefore, the giant tiger prawns were subjected to two drying treatments (*n =* 15) in order to determine if there was an observable difference in detection with varying degrees of wetness. Ten prawns were cooked and five were raw to evaluate whether the cooking style contributed to deviations in the readings; cooked samples had a drier quality compared to uncooked samples. On their lateral right side, the prawns were first blotted dry with a paper towel and then analyzed with Vanta. They were then more thoroughly dried with a heat gun (10 cm above the sample and in a slow stroking motion, the samples were exposed to 40 °C at 10 s intervals for a total of 60 s) and again subjected to analysis. These two drying treatments were considered reasonable levels of intervention for an industry-orientated protocol, and any further intervention would create an undesirable extension in the protocol’s length. The elements’ concentrations for the two treatments were compared in non-parametric paired Wilcoxon tests with R version 4.1.2 to determine if the two treatments had different effects on the elements’ quantifications (*p*, where α = 0.05).

### 2.3. Preliminary Provenance Modelling

Authenticating the provenance is one foreseeable use of this study’s soft tissue pXRF protocol. In this preliminary assessment, we aimed to see how accurately provenance could be determined with SEP via pXRF. A total of 40 tiger prawn samples collected from 6 locations/sites, including farmed and wild-caught origins, cooked and uncooked/raw were analyzed through Vanta for their SEP. Previous provenance studies have used a number of different modeling methods to determine the provenance, such as principal component analysis, partial least squares, stepwise discriminant analysis, k-nearest neighbor, canonical discriminant analysis, linear discriminant analysis (LDA), and random forest (RF) [16,19,36,37]. As the objective of this study was to establish the analytical methodology and to test if the results could be used to discriminate between the origins of *P. monodon*, we used two unsupervised dimensional reduction techniques and two supervised classification techniques. For the unsupervised analyses, nonmetric multidimensional scaling (NMDS [38]), and principal component analysis (PCA [39]) were used. For the supervised classification, random forest (RF [25]) and linear discriminant analysis (LDA [24]) were used. Supervised classification was used to test the extent to which harvest sites could be predicted. In addition, a semi-supervised method, canonical discriminant analysis (CDA [40]), was used for an additional exploratory analysis. The statistical analyses were undertaken using the R statistical package [41]. 

Data pre-treatment was also undertaken. This included scaling, where values of each element were cantered at zero, by subtracting the mean and dividing by the standard deviation. Further, for the supervised classification, elements with more than 80% zeros were removed (prior to scaling), which resulted in the removal of As (for which there were only 3 samples with very low values). Where two or more elements were correlated with a correlation coefficient of 0.8 or higher, only one of these elements was used. This resulted in the removal of Ca and Fe.

For the training of the supervised classification models, the dataset was subdivided into two sets: 80% of the data, for each group, was retained for training, and the remaining 20% was used for testing. This resulted in 32 samples for the training and 8 samples for testing (at least 1 sample from each of the different sites was randomly included in the test set). When training the RF model, the values of the tuning parameters mtry (number of elements sampled as candidates at each split) and ntree (number of trees) were systematically varied to obtain the best accuracy. Also, 5-fold cross-validation was used to determine the accuracy.

## 3. Results

### 3.1. Protocol Methodology

#### 3.1.1. Calibration and Arrangement of the Prawn Elemental Profile

The calibration factors for the *P. monodon* analysis are presented in Table 2. P, S, Cl, K, Ca, Mn, Fe, Ni, Cu, Zn, As, Sr, Zr, and Th were sufficiently quantified with the calibrated instrument, and their concentrations together created the prawn elemental profile. Zr and Th were included in the arrangement, because they were detected in the whole prawn samples with Vanta’s default settings; however, they did not have a corrective calibration factor determined with the IBA (as their concentrations were below the detection limits for the IBA). P, S, Cl, K, and Ca were the most abundant elements, with concentrations greater than 1000 ppm; the remaining elements had concentrations less than 100 ppm. The exposure time and repeatability assessments utilized only the P, S, Cl, K, and Ca concentrations, as these major elements presented clear trends. The sample moisture assessment made use of the whole profile.

#### 3.1.2. Exposure Time

**Figure 1:**
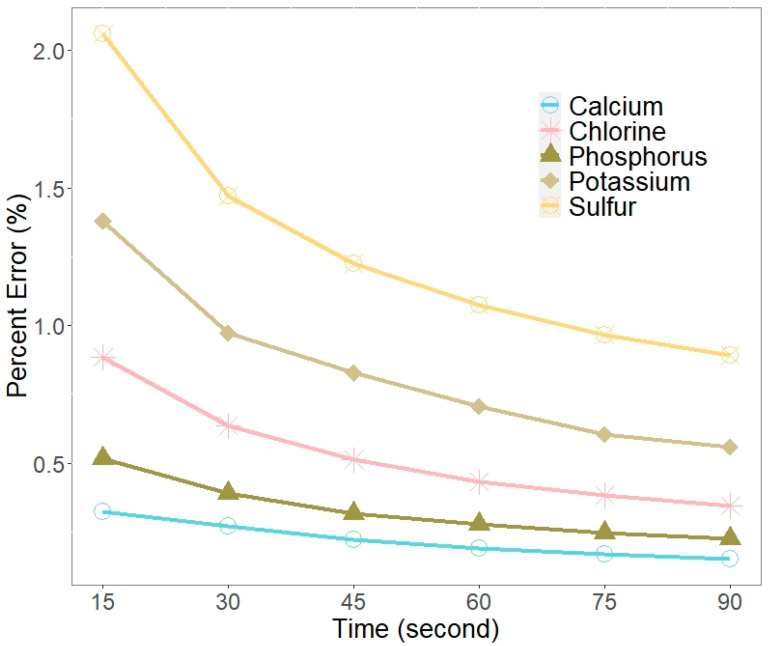
Percent error associated with Vanta pXRF exposure time for the five major elements in *P. monodon* abdominal tissue.

The percent error decreased with an increase in exposure time for P, S, Cl, K, and Ca (Figure 1). The rate of change in the slopes for all five major elements became insignificant (less than 33%) between the third and fourth slopes. Sixty seconds was the point between slope 3 and 4 and therefore the optimal exposure time; this finding was in accordance with Olympus’ recommend minimum exposure time. Users can therefore expect to conduct a pXRF analysis of *P. monodon* in 3 min when using Vanta’s three beams.

#### 3.1.3. Sample Moisture

The differences in the elemental compositions between the two moisture treatment methods were conducted separately for the cooked and raw samples, which were insignificant for all elements (Table 3). In the case of the raw prawns, the *p*-values were lower for P, Cl, and Ca (0.06). When comparing the two treatments using the cooked prawn samples only, the differences between treatments became insignificant for all the elements. This may indicate that cooking the prawns made the profile more stable, and by contrast, a raw sample’s profile might be less static because of its high moisture content. 

#### 3.1.4. Repeatability

The majority of the samples presented positive delta values (see Section 2.2.1) for the five major elements. Negative delta values presented twice for the S (−2.8 and −1.8) and Ca (−1.0 and −1.0) readings and once for Cl (−6.3) and K (−0.7). These negative delta values represented unexplained error that amounted to, at most, 6.3 ppm, which might be considered negligible when the concentrations were greater than 1000 ppm. 

**Figure 2:**
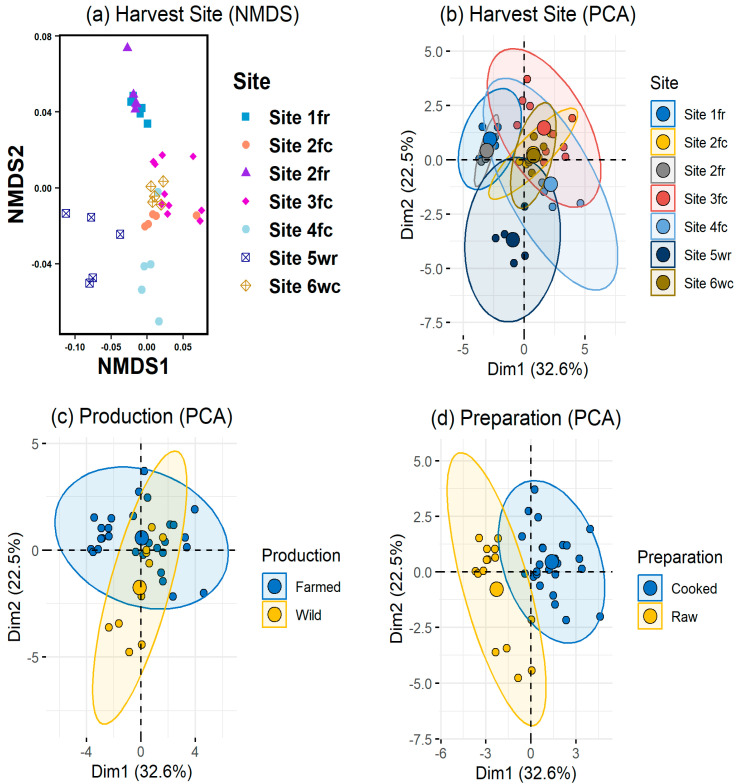
(**a**) Nonmetric multidimensional scaling (NMDS) by harvest site, (**b**) principal component analysis (PCA) by harvest site, (**c**) PCA by production (i.e., farmed, or wild), and (**d**) PCA by preparation (i.e., raw or cooked). The “f” and “w” after the site numbers in (**a**,**b**) indicated farmed or wild, respectively, and “r” and “c” indicated raw or cooked, respectively.

In Figure 2a, the cooked prawns from the separate sites were closer together than the raw prawns. A similar trend was also seen in the PCA by site (Figure 2b). This was supported by the almost separate groupings of the raw and cooked prawns in Figure 2d. The distinction between farmed and wild was not as clear, although only a small number of samples overlapped (Figure 2c).

**Figure 3:**
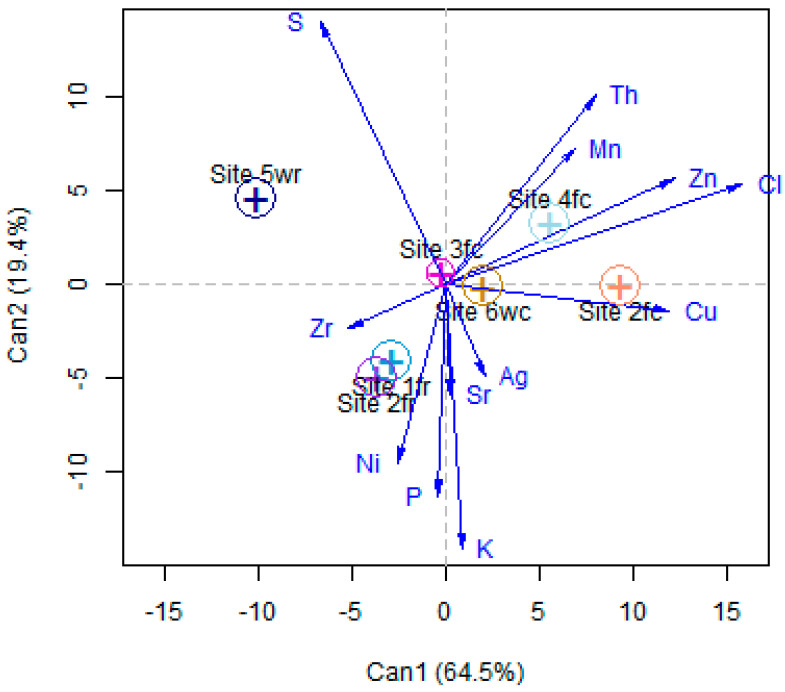
Canonical discriminant analysis results.

The groupings became clear when we considered the CDA (Figure 3). The *x*-axis results split the samples between raw and cooked prawns. The cooked samples had higher values of Cl, Zn, Th, Cu, and Mn, while the raw samples had higher concentrations of Zr. Then, the *y*-axis resulted in the splitting of the raw prawns between farmed and wild, with wild having a higher concentration of S and the farmed a higher concentration of Ni. The *y*-axis did not contribute a significant amount to the split of the cooked samples between farmed and wild.

**Figure 4:**
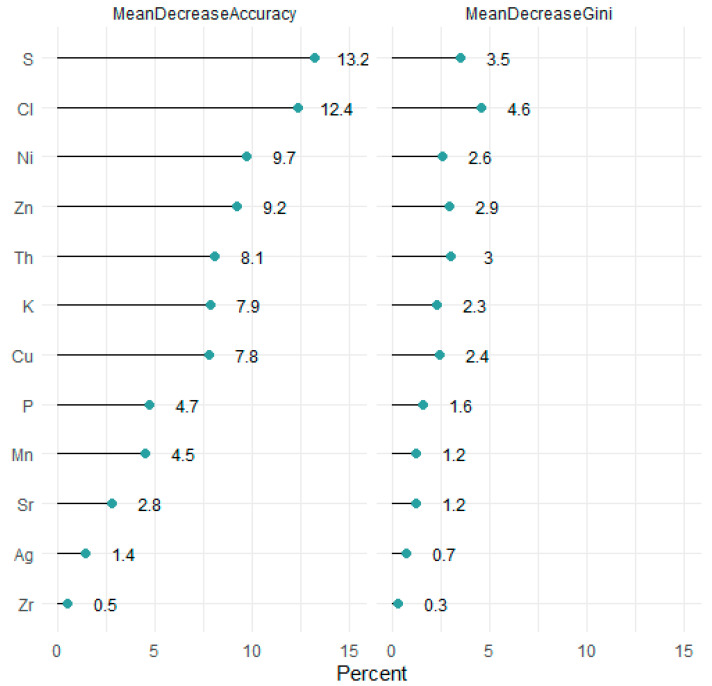
Accuracy and Gini index from the random forest model, which indicate the importance of each element in distinguishing between the sites.

The best-performing RF model, with 81% accuracy, was with *ntree* of 600 and *mtry* of 3. The importance of the elements in the classification are presented in Figure 4, indicating that S and Cl were the most important elements, followed by Ni, Zn, Th, K, and Cu. The reminder elements were less important. During the training process, misclassifications occurred between Site 1fr and Site 2fr. This was supported by the NMDS plot in Figure 2a and the CDA analysis plot in Figure 3, where there was overlap between samples from these two sites. Samples from Site 4fc were also misclassified, which again could be seen in Figure 2a that the samples from Site 4fc were more spread out, and there was overlap with the samples for Site 6wc. For the test set, only one misclassification resulted for a sample from Site 6wc, which was classified as being from Site 3fc. Figure 2a and Figure 3 show some overlap of samples from these two sites.

The LDA model also resulted in 81% accuracy of the training data, again with confusion between the samples from Site 1fr and Site 2fr. Half of the samples from Site 4fc were misclassified as being from Site 6wc. However, for the LDA, the sites for all the samples in the test set were correctly predicted.

## 4. Discussion

A biological analysis with pXRF is newly established [27,28,29,30,31], but the novelty of the present study is that a proteinaceous matrix is analyzed with satisfaction in a short period of time and with very little processing. Portable XRF-based SEP presents a viable mode for seafood industry stakeholders to profile their large and continual supply of products. Machine learning on the elemental profiles obtained via pXRF analysis resulted in 81% accuracy. Though the protocol methodology outlined in this study is specific to *Penaeus monodon,* it can be used to streamline the development of pXRF protocols for other seafood species significant to the industry, as well as other biological media.

Moisture diffracts both primary and secondary X-rays, and this scattering causes misleading detections. Two drying techniques (paper towel and heat gun) were used to determine their influence in the elemental measurement of raw and cooked prawns. No significant differences between the drying techniques were found. The findings mostly imply that either technique can be used. However, towel drying is recommended to save time with the market-level provenance application of pXRF [32]. 

The properties of the soft tissue matrix remain a source of incertitude, and because those cannot be changed for industry purposes, improvement of the pXRF application lies in better instrument calibration. As aforementioned, Vanta was calibrated using powdered prawn samples to keep in line with the IBA protocols [35]. Sacrificing the integrity of the matrix for the reputability of the IBA means the calibration is not entirely suited for the analysis of whole prawns that are less dense and have a higher moisture content compared to powders. However, counts as determined by XRF instrument spectra (i.e., semi-quantitative data that is proportional to the element concentration in the sample) are sufficient for provenance studies (e.g., [12,23]). Hence, a modification of Gadd et al.’s [14] protocols was executed in this study without compromising the soft tissue matrix. A Vanta–Itrax–IBA comparison of both powdered and unprocessed prawns may offer a solution to the calibration issue. Improved calibrations should be sought particularly for minor elements Mn, Fe, Zn, As, Zr, and Th, as they experience variability throughout the study [42] and/or play important roles in the outcomes of provenance prediction models. 

Of the range of elements in the prawn profiles, it was a combination of both major and minor elements that influenced the accuracy of the random forest provenance prediction models. In other traceability studies, the accurate and robust determination of provenance has been achieved through the combination of minor and major elements [12,43]. Addressing the multi-faceted question of provenance has hence benefited from the extensive information captured by this multielement analysis, regardless of the instrument. A direct relationship has yet to be understood for each element in the prawn profile, but some relationships may be inferred. Cl and S played important roles in accuracy for the random forest models. The saline qualities of the prawns’ farmed and wild-caught origins and geographic locations are assumed to influence their Cl measurements [12], but the concentrations in food may also be a function of post-harvest treatments like boiling [44]. Other elements like K are also greatly affected by wet cooking methods [45], and soaking food products to lower the K levels is even a suggested practice for those suffering from hyperkalemia [46]. 

Moreover, this brings attention to the role cooking plays in the elemental composition of seafood products. Abd-Elghany et al. [47] found that the cooking of prawns and crabs (seafood products) reduced the contents of some elements. A multielement analysis most likely can help to overcome this issue, as the model predictions are related to the compositions of various elements instead of a few elements [1,12,16,48]. To reiterate, three of the eight prawn groups in the models consisted of uncooked samples. An uncooked sample was involved in each instance where random forest was not able to accurately assign a sample to its group. 

As for the effects of cooking on the profiles themselves, As was only able to be detected in the uncooked samples. This observation has several possible explanations; it could be that the aberrant −21.486 correction factor misinformed Vanta’s readings. Another possibility is that an excess of water interfered with the detection of As, as is often observed in humid soil samples [49]. Boiling meat causes water to move against the moisture gradient and out of the proteinaceous matrix, causing the networks to shrink and become denser [50]. Therefore, the moisture contents of cooked and uncooked seafood products are different, and this will affect detection of As and other elements. On the other hand, boiling food is thought to increase the As burden of seafood products, especially if the water is contaminated [51], and it is also worth noting that the repeated freezing and thawing of the prawns throughout the course of the study also changed the samples’ water retention each time [52]. Understanding the role of cooking and storage is therefore important for pXRF quality assurance measurements pertaining to heavy metal contamination.

## 5. Conclusions

Portable XRF has proven to be a potential rapid and reliable analysis for elemental detection and quantification in a biological medium. Seafood elemental profiling via pXRF analysis determines the provenance with high accuracy (>80%) when implemented in machine learning models. A lack of density and high moisture content in the soft tissue matrix, along with cooking style, influence the composition and detection independently. The calibration factors that correct for wetness and cooking improve the pXRF protocol and make it better suited to the realities and needs of the seafood industry. Future research should consider comparing pXRF with other non-destructive analysis methods similar to XRF, such as FTIR, and Raman for the provenance and the determination of the geographical origins. Furthermore, additional testing of the methodology on the matrices of different seafood products will allow the pXRF to be used to determine their provenance.

## Figures and Tables

**Table 1:** *P. monodon* provenance groups, their harvest site and state, production method, cooking information, and date of collection.

Site	State	Production	Cooking	Collection Date
1fr	Queensland	Farmed	Uncooked	21 February
2fr	Queensland	Farmed	Uncooked	21 February
2fc	Queensland		Cooked	21 February
3fc	Queensland	Farmed	Cooked	21 January
3fc	Queensland		Cooked	21 February
4fc	Queensland	Farmed	Cooked	21 January
5wr	New South Wales	Wild-caught	Uncooked	21 January
6wc	New South Wales	Wild-caught	Cooked	21 January

**Table 2:** *P. monodon* elemental profile arrangement and correction factors for the Vanta analysis.

Element	Correction Factor
*P*	0.7196
*S*	0.3138
*Cl*	0.6894
*K*	0.7977
*Ca*	1.1036
*Mn*	2.2247
*Fe*	0.4202
*Ni*	−1.625
*Cu*	0.4114
*Zn*	0.1765
*As*	−21.486
*Sr*	1.3978
*Zr*	N/A
*Th*	N/A

**Table 3:** Wilcoxon test *p*-values comparing element concentrations for towel and heat gun drying treatments (* indicates that all samples in this group had As values of zero).

Element	*p*-Value	
	Raw	Cooked
P	0.06	0.92
S	0.31	0.85
Cl	0.06	0.77
K	0.31	0.49
Ca	0.06	1.00
Mn	0.20	0.10
Fe	0.19	0.17
Ni	1.00	0.18
Cu	0.77	0.63
Zn	0.09	0.15
As	0.35	*
Sr	0.77	0.92
Zr	0.35	0.77
Th	1.00	0.09

## Data Availability

The datasets presented in this manuscript are not readily available due to commercial-in-confidence. Requests to access the datasets should be directed to the corresponding author.
